# Exploring the genetics of lesion and nodal resistance in pea (*Pisum sativum* L.) to *Sclerotinia sclerotiorum* using genome‐wide association studies and RNA‐Seq

**DOI:** 10.1002/pld3.64

**Published:** 2018-06-26

**Authors:** Hao‐Xun Chang, Hyunkyu Sang, Jie Wang, Kevin E. McPhee, Xiaofeng Zhuang, Lyndon D. Porter, Martin I. Chilvers

**Affiliations:** ^1^ Department of Plant, Soil and Microbial Sciences Michigan State University East Lansing Michigan; ^2^ Department of Plant Biology Michigan State University East Lansing Michigan; ^3^ Department of Plant Sciences and Plant Pathology Montana State University Bozeman Montana; ^4^ Department of Horticulture and Crop Science The Ohio State University Wooster Ohio; ^5^ USDA‐ARS Prosser Washington

**Keywords:** genome‐wide association study, glutathione S‐transferase, lesion resistance, nodal resistance, pea (*Pisum sativum* L.), RNA‐Seq, white mold (*Sclerotinia sclerotiorum*)

## Abstract

The disease white mold caused by the fungus *Sclerotinia sclerotiorum* is a significant threat to pea production, and improved resistance to this disease is needed. Nodal resistance in plants is a phenomenon where a fungal infection is prevented from passing through a node, and the infection is limited to an internode region. Nodal resistance has been observed in some pathosystems such as the pea (*Pisum sativum* L.)‐*S. sclerotiorum* pathosystem. In addition to nodal resistance, different pea lines display different levels of stem lesion size restriction, referred to as lesion resistance. It is unclear whether the genetics of lesion resistance and nodal resistance are identical or different. This study applied genome‐wide association studies (GWAS) and RNA‐Seq to understand the genetic makeup of these two types of resistance. The time series RNA‐Seq experiment consisted of two pea lines (the susceptible ‘Lifter’ and the partially resistant PI 240515), two treatments (mock inoculated samples and *S. sclerotiorum*‐inoculated samples), and three time points (12, 24, and 48 hr post inoculation). Integrated results from GWAS and RNA‐Seq analyses identified different redox‐related transcripts for lesion and nodal resistances. A transcript encoding a glutathione S‐transferase was the only shared resistance variant for both phenotypes. There were more leucine rich‐repeat containing transcripts found for lesion resistance, while different candidate resistance transcripts such as a VQ motif‐containing protein and a myo‐inositol oxygenase were found for nodal resistance. This study demonstrated the robustness of combining GWAS and RNA‐Seq for identifying white mold resistance in pea, and results suggest different genetics underlying lesion and nodal resistance.

## 1 INTRODUCTION

*Sclerotinia sclerotiorum* (Lib.) de Bary, the causal agent of white mold disease, is one of the most destructive plant pathogens worldwide. *S. sclerotiorum* is capable of infecting more than 400 host plants and causes millions of dollars of crop yield losses each year (Bolton, Thomma, & Nelson, 2006). Several studies have reported different secondary metabolites, effectors, and pathogenicity factors of *S. sclerotiorum* that are involved in establishing the infection (Bolton et al., 2006; Mbengue et al., 2016; Wei & Clough, 2016). One of the well‐known virulence strategies is the production of oxalic acid, which creates a low pH and acidic environment for infection (Xu, Xiang, White, & Chen, 2015). Oxalic acid suppresses reactive oxygen species (ROS) produced by plants at the beginning of infection and generates a reducing status that favors colonization (Williams, Kabbage, Kim, Britt, & Dickman, 2011). Fine‐tuned redox homoeostasis from the initial reducing status to the later oxidative status in plant tissues is important for *S. sclerotiorum* to switch from the initial biotrophic lifestyle to the later necrotrophic lifestyle (Kabbage, Yarden, & Dickman, 2015). Studies searching for plant resistance to *S. sclerotiorum* have found quantitative interactions (McCaghey et al., 2017), and potential resistance genes included those with functions to maintain ROS and redox stresses during *S. sclerotiorum* infection (Girard et al., 2017; Ranjan et al., 2017; Zhou, Sun, & Xing, 2013).

Pea (*Pisum sativum* L.) is an important legume crop in the United States (Tayeh et al., 2015), and white mold continuously causes substantial damage and yield reduction (Biddle, 2001). *Sclerotinia sclerotiorum* infection begins when ascospores of *S. sclerotiorum* colonize blooms and invade through petioles into the stem. Severely infected plants will wilt and lodge. Resistance to white mold in pea has been observed via two different phenotypes. The first is lesion size where the length of stem lesion is measured after inoculation. The second phenotype is referred to as nodal resistance, which appears to be a unique mode of resistance and has been observed in some varieties of pea and soybean (Calla, Voung, Radwan, Hartman, & Clough, 2009; Porter, 2011; Porter, Hoheisel, & Coffman, 2009). Nodal resistance can be defined as the inhibition of lesion expansion at a node limiting pathogen colonization of plant stem tissue. Restriction of lesion expansion at the nodes has also been observed for stem‐infecting fungi such as *Diaporthe* and *Macrophomina* species on soybean and cowpea (Hobbs, Schmitthenner, & Ellett, 1981; Muchero, Ehlers, Close, & Roberts, 2011). However, nodal resistance has been rarely documented and other than knowing lignin content is negatively correlated with nodal resistance in soybean (Peltier, Hatfield, & Grau, 2009), our understanding is limited.

Transcriptomics and differential expression (DE) analysis using RNA‐Seq have become a standard approach to identifying resistance genes for white mold, and studies have applied this approach to oilseed rape (*Brassica napus*) and pea (Girard et al., 2017; Seifbarghi et al., 2017; Zhuang, McPhee, Coram, Peever, & Chilvers, 2012). While most of these studies focused on the expression comparisons between a resistant and a susceptible variety, the genetic diversity of white mold resistance in *B. napus* might be underestimated using only this approach. Genome‐wide association study (GWAS) is a robust approach to map white mold resistance and to capture the resistance diversity in a germplasm collection (Moellers et al., 2017; Wei et al., 2016, 2017). The GWAS approach has been demonstrated in soybean (*Glycine max* Merr. L.) resistance to *S. sclerotiorum* where numerous single nucleotide polymorphisms (SNPs) associated with this quantitative resistance were discovered (Bastien, Sonah, & Belzile, 2014; Moellers et al., 2017; Wen et al., 2018; Wu, Zhao, Liu, et al., 2016). However, mapping results may discover SNPs that locate in intergenic genomic regions, and the interpretation of a confidence interval relies on the size of linkage disequilibrium (Bush & Moore, 2012). RNA‐Seq and GWAS both have their advantages, and combining them provides a powerful tool to discover not only active genes that express in response to treatments, but also genetic diversity and SNPs associated with the treatment. This combined strategy has been applied to understand white mold resistance and yields in *B. napus* (Lu et al., 2017; Wei et al., 2016) and soybean (Wen et al., 2018), but not in pea. Because genes that can be found by both GWAS and RNA‐Seq will have higher potential in contributing to white mold resistance, this study aimed to understand and compare the genetics of lesion and nodal resistance by applying both GWAS and RNA‐Seq approaches in the pea‐*S. sclerotiorum* pathosystem.

## 2 MATERIALS AND METHODS

### 2.1 GWAS: data source and analysis

Data used for GWAS were published in Porter et al. (2009). Briefly, there were 282 pea lines with a mean lesion resistance rating. The white mold fungus (*S. sclerotiorum*) Scl02‐05 isolated from pea in Quincy, Washington, USA in 2003 was used for inoculations (Porter et al., 2009). A mean lesion resistance was measured in centimeters of lesion size, smaller values representing higher resistance. The data were collected after 72 hpi in a humid greenhouse and day/night temperature ranges around 28°C/15°C. There were 266 pea accessions with nodal resistance ratings. Nodal resistance was measured using an ordinal scale from 0 to 5 after 2 weeks postinoculation, where 0 = dead plant; 1 = lesion expanded down the stem from the fourth inoculated node to the first node; 2 = lesion expanded from the fourth to the second node; 3 = lesion expanded from the fourth node to the third node; 4 = lesion did not expand beyond the initial inoculation point at the fourth node (Porter et al., 2009). There were four to eight replications to represent each accession. The USDA Pea Single Plant Plus Collection with SNP data was included in this study (Holdsworth et al., 2017). Association test was conducted in PLINK version 1.9 (Purcell et al., 2007). Population stratification was controlled using a pairwise identity‐by‐state (IBS) clustering with a maximum clustering node of 2 and a *p* value cutoff of 0.05 for the pairwise population concordance test. The IBS clustering matrix was included in a basic association test, and a minor allele frequency of 0.05 was applied. The empirical *q* value at 0.01 from an adaptive permutation test with default parameters was used to determine association significance. The genotyping‐by‐sequencing (GBS) raw reads containing significant SNPs were searched against the Trinity de novo transcriptome (assembled in the following sections) using BLASTN to acquire annotations at an *E* value cutoff of 10^−5^.

### 2.2 Plant inoculations for RNA‐Seq

A white mold‐susceptible pea (*P. sativum* L.) cultivar ‘Lifter’ (PI 628276) and a white mold‐partially resistant pea accession, PI 240515, were used in this study. These two lines were also used as parents in the development of a recombinant inbred line for investigating resistance, in a separate study. The same *S. sclerotiorum* isolate Scl02‐05 was used for RNA‐Seq experiments. Seeds from ‘Lifter’ and PI 240515 were planted at a depth of 1 cm in pasteurized soil in a plastic pot (approximately 170 cm^3^). The soil consisted of a mixture of 85 L of Special Blend Soil Mix (Sun Gro Horticulture, Bellevue, WA), 113 L of propagation‐grade coarse perlite (Supreme Perlite Company, Portland, OR), and 900 g of Scotts Osmocote Classic 14‐14‐14 (The Scotts Company, Marysville, OH). Plants were grown in a growth chamber at 23°C/20°C (day/night), with a photoperiod of 14 hr and 170 μmol quanta (s^−1 ^m^−2^) for 2 weeks. One day before *S. sclerotiorum* inoculations, pea plants were covered with a thick transparent plastic cover, which filtered the amount of light reaching the plants down to 45–55 μmol quanta (s^−1 ^m^−2^), and maintained a high humidity (RH %; 86.91 ± 13.45; WatchDog 1000 Series, Spectrum Technologies Inc., Aurora, IL). Pea plants were inoculated at the fourth node leaf axil with a 49 mm^3^ *S. sclerotiorum* colonized agar plug from the leading edge of a culture grown on potato dextrose agar (PDA; BD Company, Sparks, MD). Mock inoculations were performed with sterile PDA plugs.

### 2.3 RNA extraction and sequencing

The RNA‐Seq experiment had a time series factorial design with two varieties (‘Lifter’ and PI 240515), two treatments (mock and *S. sclerotiorum* inoculation), and three time points at 12, 24, and 48 hr postinoculation (hpi). For each condition, two biological replicates of pea samples were collected. In order to acquire RNA samples that provide both expression data for lesion and nodal resistance, tissues within 2 cm of the inoculated fourth node were collected from at least 12 plants for each biological replicate. These tissues were immediately frozen in liquid nitrogen. Total RNA was isolated using Trizol^®^ reagent (Invitrogen, Carlsbad, CA) according to manufacturer's instructions. DNase digestion (Promega, Madison, WI) was performed on the RNA extract to remove potential DNA contamination. RNA samples were further purified using the RNeasy Plant Mini Kit (Qiagen, Valencia, CA) and quality verified using a 2100 Bioanalyzer RNA Nanochip (Agilent, Santa Clara, CA). Samples achieved an RNA integrity number (RIN) value above 7.5 and were quantified using the Qubit^®^ 2.0 Fluorometer (Invitrogen), and a total of 10 μg RNA were used for cDNA library preparations following the Illumina TruSeq RNA Preparation Kit manufacturer's instructions (Illumina, San Diego, CA). A paired‐end 2 × 75 base sequencing was run on the Illumina GA IIx sequencer (Illumina) at the Research and Technology Support Facility at Michigan State University.

### 2.4 De novo transcriptome assembly

Illumina raw reads were quality checked using FastQC version 0.11.5 (Andrews, 2010) and quality controlled using FASTX‐toolkit version 0.0.14 (Gordon, 2014). Reads with 90 percent length above Phred score 30 were kept for analyses. Trimmomatic version 0.33 was used in default mode to remove adapters and to separate paired reads and single reads (Bolger, Lohse, & Usadel, 2014), and only paired reads were used for de novo assembly. All samples were pooled and aligned to the complete nearly gapless *S. sclerotiorum* genome sequence (Derbyshire et al., 2017) using the sensitive mode of Bowtie2 version 2.2.6 and Tophat2 version 2.1.0 (Kim et al., 2013). Reads unmapped to *S. sclerotiorum* genome were de novo assembled by Trinity version 2.4.0 using K‐mer size 25, 29, and 32 (Grabherr et al., 2011; Haas et al., 2013).

### 2.5 Differential expression, heatmap clustering, and gene ontology (GO) analyses

A k‐mer index of 31 bp was built for the Trinity de novo transcriptome and paired‐end reads were pseudo‐aligned to the index using Kallisto version 0.43.0 with 1,000 bootstrap (Bray, Pimentel, Melsted, & Pachter, 2017). DE analysis was conducted using Sleuth version 3 in default mode using transcripts per million (TPM) normalization (Bray et al., 2017). The default filter setting was applied such that transcripts with more than 5 estimated counts in 47 percent of samples were kept for DE analysis. The principal component analysis (PCA) was used to visualize variation structure among all samples, and the hierarchical clustering using the “stats” package version 3.2.1 in R. The Ward's D2 method was applied to group transcripts in the heatmap analysis (Murtagh & Legendre, 2014). A time series model with three explanatory variables, including the variety (‘Lifter’ and PI 240515), the treatment (mock and *S. sclerotiorum* inoculation), and the time (12, 24, and 48 hpi), were included in a full model whereas a reduced model excluded a variable of interest. A model comparison using likelihood ratio test was used to identify transcripts with DE in response to the variable of interest, and a multiple comparison‐corrected *q* value at 0.05 was used to determine the significance. De novo transcripts were functionally annotated by soybean coding sequences using BLASTN at an *E* value cutoff of 10^−5^, and soybean gene models with orthologous de novo transcripts of pea were subjected to agriGO v2.0 singular enrichment analysis (SEA) using Fisher's exact test with Yekutieli correction to control false discovery rate (FDR) at 0.05 in multiple‐tests (Tian et al., 2017).

### 2.6 Expression verification using reverse transcription quantitative polymerase chain reaction (RT‐qPCR)

A factorial experiment was set up with two pea varieties (‘Lifter’ and PI 240515), two treatments (mock and *S. sclerotiorum* inoculation), three time points (12, 24, and 48 hpi), and three biological replicates for each factorial combination. RNA samples were extracted following the standard procedures of Direct‐zol^™^ RNA MiniPrep Plus Kit (Zymo Research, Irvine, CA). cDNA were synthesized using SuperScript^®^ III First‐strand Synthesis System (Thermo Fisher Scientific, Waltham, MA) with random primers (Promega). Four candidate genes were selected for verification, including the glutathione S‐transferase (forward: 5′‐GTG ATG CTC ATT CCG GTT CT, reverse: 5′‐TGT TTG GCC TCC CAG TTA TG), myo‐inositol oxygenase (forward: 5′‐GAA TTT GAA GTG GCT CCA TGT ATT T, reverse: 5′‐GCG AGA GAT AAT ACG GCT TCA C), VQ‐containing protein (forward: 5′‐TGG CTC AGC AAC TTC AGA AT, reverse: 5′‐CCA CAA CCA ATC CAT CAG AAA C), ACT domain repeat protein (forward: 5′‐GGA TCG TCC TAA GTT GCT GTT, reverse: 5′‐TGT TCT GCT ATG GGA CTG TTG). The expressions were normalized to a pea reference gene β‐tubulin (forward: 5′‐GCT CCC AGC AGT ACA GGA CTC T, reverse: 5′‐TGG CAT CCC ACA TTT GTT GA) (Die, Román, Nadal, & Gonzálex‐Verdejo, 2010) and to the expression level of ‘Lifter’ mock samples at 12 hpi. RT‐qPCR was conducted using the *Power*SYBR Green PCR Master Mix with two technical replicates for each biological replicate. The StepOne Plus Real‐Time PCR System (Thermo Fisher Scientific) was used and samples with a *C*_t_ value above 40 were regarded as undetectable missing data points in downstream statistical analysis. The response variables in the ANOVA test were the power transformed −∆∆*C*_t_ values using the Box–Cox method to fulfill normality and equal variance assumptions. The explanatory variables are the factorial combination of two varieties, two treatments, and three time points, and the significance was detected using Tukey's HSD at *p* value of 0.05.

## 3 RESULTS

### 3.1 Phenotype data for lesion and nodal resistance

**Figure 1:**
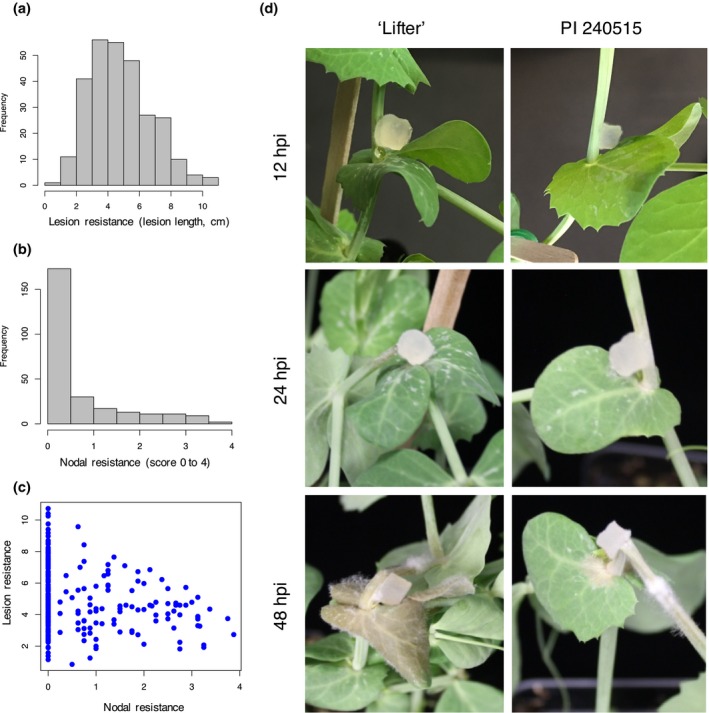
Lesion and nodal resistance phenotypes of pea lines used in genome‐wide association studies, and phenotypes of ‘Lifter’ and PI 240515 used in RNA‐Seq. (a) Phenotypic distribution of lesion resistance (lesion size in centimeter). (b) Phenotypic distribution of nodal resistance (score 0 = dead plant, 4 = lesion restricted to the inoculated node number 4). (c) Pearson's correlation between lesion and nodal resistance demonstrates slight but significant negative correlation (−0.19, *p *<* *0.05; negative due to the inverse rating scale for nodal resistance). (d) Phenotypic difference between a susceptible cultivar ‘Lifter’ and a partially resistant accession PI 240515 over time. A potato dextrose agar block containing actively growing hyphal tips of *Sclerotinia sclerotiorum* was used for inoculation. PI 240515 has partial resistance and displays slower disease progress compared to susceptible ‘Lifter.’ Infection and damping‐off can be observed in ‘Lifter’ as early as 12 and 24 hpi, respectively, but not PI 240515. Infection expands in “Lifter” as early at 48 hpi, and infection can be observed around the inoculated site of PI 240515 at 48 hpi

There were 282 and 266 pea germplasm lines screened for lesion and nodal resistance, respectively (Supporting Information Table S1). Lesion resistance was measured by recording lesion size in centimeters at 72 hpi, where a smaller value indicated greater lesion resistance. Nodal resistance was measured by recording which node the *S. sclerotiorum* infection expanded to (1–4 represented the first to the fourth node, where 0 indicated a completely dead individual with a lesion to the soil line). A larger value indicated a greater nodal resistance scored 2 weeks postinoculation. While the lesion resistance distribution approximated a normal distribution (Figure 1a), the nodal resistance distribution was highly skewed toward zero with only 12 germplasm lines rated with a score above 3 (Figure 1b). There was a slight but significant correlation between lesion and nodal resistance (Pearson's correlation coefficient: −0.19, *p *<* *0.05). The correlation was negative due to the inverse disease scales between lesion size and nodal resistance rating. There were lines that demonstrated a slow lesion progression on the stem, but the lesion expanded and killed the plant after 2 weeks. On the other hand, there were lines that demonstrated a larger stem lesion initially after 72 hpi, but the lesion was arrested at a subsequent node, indicating the possibility of different genetic mechanisms of these two types of resistances (Figure 1c). White mold susceptible line ‘Lifter’ and the white mold partially resistant line PI 240515 were selected for RNA‐Seq. PI 240515 displays not only a slower disease progress compared to the susceptible ‘Lifter’ under growth chamber conditions (Figure 1d), but also a better resistance performance in field trials (McPhee & Muehlbauer, 2002; Zhuang, McPhee, Coram, Peever, & Chilvers, 2013). These two lines were also used as parents for the development of recombinant inbred mapping population. A time course RNA‐Seq experiment was set up in a factorial design, and a total of 12 samples with about 700 million reads were acquired from Illumina sequencing (Supporting Information Table S2).

### 3.2 Genome‐wide association study

A total of 35,658 SNPs were included in the association analysis using PLINK (Purcell et al., 2007). There were 206 and 118 SNPs found to be significantly associated with lesion and nodal resistance, respectively (Supporting Information Table S3 and S4, respectively). Without a standard genome for pea, the position and chromosome information for SNPs were deficient and it also made the annotation to these SNPs difficult. In order to understand the annotations of these significant SNPs, the original GBS raw reads harboring each SNP were retrieved (Holdsworth et al., 2017) and searched against our RNA‐Seq de novo transcriptome using BLASTN.

### 3.3 De novo transcriptome assembly

Using a stringent quality control threshold that keeps only raw reads with 90 percent of bases above Phred score of 30 (error rate 0.001%, one error per thousand bases), paired‐end reads were mapped to the complete nearly gapless *S. sclerotiorum* genome using Tophat2 (Derbyshire et al., 2017; Kim et al., 2013). The de novo transcriptome using k‐mer of 29 bp, which contains 96,588 transcripts including isoforms, resulted in the highest assembly quality (Table 1) similar to a previous de novo transcriptome of pea (Kerr, Gaiti, Beveridge, & Tanurdzic, 2017), and the re‐mapped rate at 80% was satisfactory based on an empirical threshold of Trinity (Grabherr et al., 2011; Haas et al., 2013). Accordingly, the k‐mer 29 de novo transcriptome was selected and a total of 60,598 transcripts were extracted from the longest representative isoform per gene model in the k‐mer 29 de novo transcriptome (Table 1).

**Table 1:** Quality assessment of Trinity de novo transcriptome assemblies

Assembly statistics	K‐mer 25	K‐mer 29	K‐mer 32
Total number of transcripts^a^	104,743	96,588	89,213
Transcript contig N50 (bp)	1,821	1,891	1,879
Mean length (bp)	1,120.68	1,158.61	1,139.89
Median length (bp)	746	780	746
Assembled bases (bp)	117,382,962	111,907,459	101,692,986
Total number of genes^b^	63,432	60,598	59,611
Gene contig N50 (bp)	1,506	1,608	1,611
Mean length (bp)	856.40	890.64	892.03
Median length (bp)	470	473	472
Assembled bases (bp)	54,323,175	53,970,897	53,175,090
Percentage of GC	38.58	38.64	38.66
RNA representation rate (%)	79.7	80.0	80.0

*Note*

^a^Transcript statistics include isoforms. ^b^Gene statistics include only the longest transcript for all possible isoforms.

### 3.4 Localization of Significant SNPs in GWAS using de novo transcripts from RNA‐Seq

There were 206 significant SNPs associated with lesion resistance, but only 96 SNPs matched to de novo transcripts. Among these 96 de novo transcripts, 66 of them could be annotated with an orthologous soybean gene (Table 2, Supporting Information Table S3). In terms of nodal resistance, there were 118 significant associated SNPs, and 61 SNPs matched to de novo transcripts. Among these 61 de novo transcripts, 33 of them could be annotated with an orthologous soybean gene (Table 3, Supporting Information Table S4). In comparing the GWAS for lesion and nodal resistance, only one SNP (TP13557) can be found in both cases, and the de novo transcript containing this SNP was annotated as a putative glutathione S‐transferase (GST) (orthologous to soybean gene Glyma.06G117800). Together with the weak phenotype correlation, the results suggest the genetics of lesion and nodal resistance may be different.

**Table 2:** Candidate resistance transcripts identified in GWAS for lesion resistance

SNP	GWAS *q* value	Allele	MAF	De novo transcript	*E* value^a^	Gm.W82.a2.v1	Annotation
TP104551	1.41 × 10^−04^	A/G	0.108	TRINITY_DN19399_c0_g1_i2	2.00 × 10^−06^	Glyma.05G228200	Xylogalacturonan β‐1,3‐xylosyltransferase
TP82039	1.22 × 10^−03^	C/T	0.489	TRINITY_DN22733_c0_g1_i1	7.00 × 10^−25^	Glyma.16G023200	TPR‐containing protein
TP132492	1.25 × 10^−03^	C/A	0.100	TRINITY_DN11274_c0_g2_i1	2.00 × 10^−26^	Glyma.20G193300	CC‐NBS‐LRR disease resistance protein
TP176436	1.33 × 10^−03^	A/G	0.356	TRINITY_DN15345_c0_g1_i2	6.00 × 10^−19^	Glyma.19G233900	Oxidoreductase
TP192026	2.68 × 10^−03^	G/A	0.185	TRINITY_DN12885_c0_g1_i1	5.00 × 10^−26^	Glyma.04G159700	UDP‐arabinopyranose mutase
TP58726	2.81 × 10^−03^	C/T	0.145	TRINITY_DN21727_c0_g1_i1	7.00 × 10^−25^	Glyma.17G090900	U‐box/ARM repeat superfamily protein
TP46107	3.03 × 10^−03^	A/C	0.400	TRINITY_DN23674_c1_g2_i1	2.00 × 10^−26^	Glyma.03G168000	Pleiotropic drug resistance ABC transporter
59136_75	3.61 × 10^−03^	A/G	0.085	TRINITY_DN24902_c0_g1_i1	5.00 × 10^−15^	Glyma.16G130700	Serine carboxypeptidase
64833_37	3.94 × 10^−03^	T/C	0.426	TRINITY_DN21727_c0_g1_i1	1.00 × 10^−30^	Glyma.17G090900	U‐box/ARM repeat superfamily protein
TP40425	3.96 × 10^−03^	C/T	0.069	TRINITY_DN11862_c0_g1_i1	7.00 × 10^−25^	Glyma.09G242500	PPR‐containing protein
TP5714	3.97 × 10^−03^	A/G	0.160	TRINITY_DN8622_c0_g1_i2	7.00 × 10^−25^	Glyma.20G013200	U‐box/ARM repeat superfamily protein
TP122891	4.37 × 10^−03^	C/T	0.065	TRINITY_DN20767_c0_g1_i1	7.00 × 10^−25^	Glyma.06G065000	Cellulose synthase
TP107762	4.65 × 10^−03^	C/G	0.348	TRINITY_DN21987_c1_g2_i1	2.00 × 10^−19^	Glyma.08G284100	LRR‐RLK
TP105293	7.28 × 10^−03^	C/T	0.490	TRINITY_DN22449_c0_g1_i1	2.00 × 10^−26^	Glyma.13G359600	PPR‐containing protein
TP14472	7.60 × 10^−03^	A/G	0.108	TRINITY_DN23601_c0_g1_i1	2.00 × 10^−19^	Glyma.08G160900	ABC transporter
54792_76	7.91 × 10^−03^	C/G	0.321	TRINITY_DN23920_c0_g1_i14	2.00 × 10^−15^	Glyma.13G370300	Pleiotropic drug resistance ABC transporter
TP56848	9.05 × 10^−03^	A/C	0.193	TRINITY_DN23559_c0_g2_i3	2.00 × 10^−26^	Glyma.06G178400	Copper amine oxidase
67025_44	9.21 × 10^−03^	T/C	0.352	TRINITY_DN29578_c0_g1_i1	6.00 × 10^−27^	Glyma.06G131900	Cytochrome b5
TP184273	9.52 × 10^−03^	A/T	0.055	TRINITY_DN22193_c0_g1_i4	7.00 × 10^−25^	Glyma.19G004700	ARM repeat superfamily protein
TP13557	9.82 × 10^−03^	C/T	0.194	TRINITY_DN7903_c0_g1_i2	7.00 × 10^−25^	Glyma.06G117800	Glutathione S‐transferase

*Notes*

GBS: genotyping‐by‐sequencing; GWAS: genome‐wide association studies; MAF: minor allele frequency; SNP: single nucleotide polymorphism.

^a^ *E* value of BLASTN using a GBS read containing the significant SNP against the de novo transcriptome.

**Table 3:** Candidate resistance transcripts identified in GWAS for nodal resistance

SNP	GWAS *q* value	Allele	MAF	De novo transcript	*E* value^a^	Gm.W82.a2.v1	Annotation
56725_68	3.74 × 10^−03^	G/C	0.197	TRINITY_DN5298_c0_g1_i1	3.00 × 10^−30^	Glyma.05G107600	ACT domain repeat protein
TP41550	4.50 × 10^−03^	A/G	0.167	TRINITY_DN25769_c0_g1_i1	7.00 × 10^−25^	Glyma.09G051900	VQ motif‐containing protein
TP13557	4.59 × 10^−03^	C/T	0.194	TRINITY_DN7903_c0_g1_i2	7.00 × 10^−25^	Glyma.06G117800	Glutathione S‐transferase
TP52272	4.64 × 10^−03^	T/C	0.106	TRINITY_DN23515_c1_g1_i4	1.00 × 10^−15^	Glyma.12G053900	β‐glucosidase
TP164197	4.83 × 10^−03^	T/A	0.093	TRINITY_DN41476_c0_g1_i1	2.00 × 10^−26^	Glyma.08G254000	PPR repeat‐containing protein
161268_51	5.04 × 10^−03^	T/C	0.095	TRINITY_DN21874_c0_g2_i1	4.00 × 10^−35^	Glyma.04G029500	RING/U‐box superfamily protein
53592_14	6.47 × 10^−03^	C/T	0.147	TRINITY_DN21524_c0_g1_i1	4.00 × 10^−35^	Glyma.05G224500	Myo‐inositol oxygenase
TP108888	6.96 × 10^−03^	C/T	0.248	TRINITY_DN21142_c0_g2_i2	7.00 × 10^−25^	Glyma.06G183500	Protein kinase superfamily protein
TP119499	7.49 × 10^−03^	T/C	0.053	TRINITY_DN43663_c0_g1_i1	2.00 × 10^−12^	Glyma.18G202100	Calcium‐dependent protein kinase
TP163256	8.04 × 10^−03^	A/G	0.357	TRINITY_DN7950_c0_g1_i1	7.00 × 10^−25^	Glyma.07G018400	Peroxisome‐related protein
14350_13	8.25 × 10^−03^	A/G	0.124	TRINITY_DN16214_c1_g2_i1	2.00 × 10^−32^	Glyma.09G011200	Cytochrome b‐561
TP164952	9.52 × 10^−03^	T/C	0.097	TRINITY_DN23515_c1_g1_i4	2.00 × 10^−06^	Glyma.12G053900	β‐glucosidase
123212_21	9.66 × 10^−03^	G/A	0.155	TRINITY_DN21917_c0_g1_i2	2.00 × 10^−27^	Glyma.11G202700	TPR repeat‐containing protein
33803_4	9.96 × 10^−03^	C/T	0.058	TRINITY_DN23419_c1_g1_i6	4.00 × 10^−35^	Glyma.07G151800	β‐glucosidase

*Notes*

GBS: genotyping‐by‐sequencing; GWAS: genome‐wide association studies; MAF: minor allele frequency; SNP: single nucleotide polymorphism.

^a^ *E* value of BLASTN using a GBS read containing the significant SNP against the de novo transcriptome.

### 3.5 Principal component analysis (PCA) of RNA‐Seq samples

**Figure 2:**
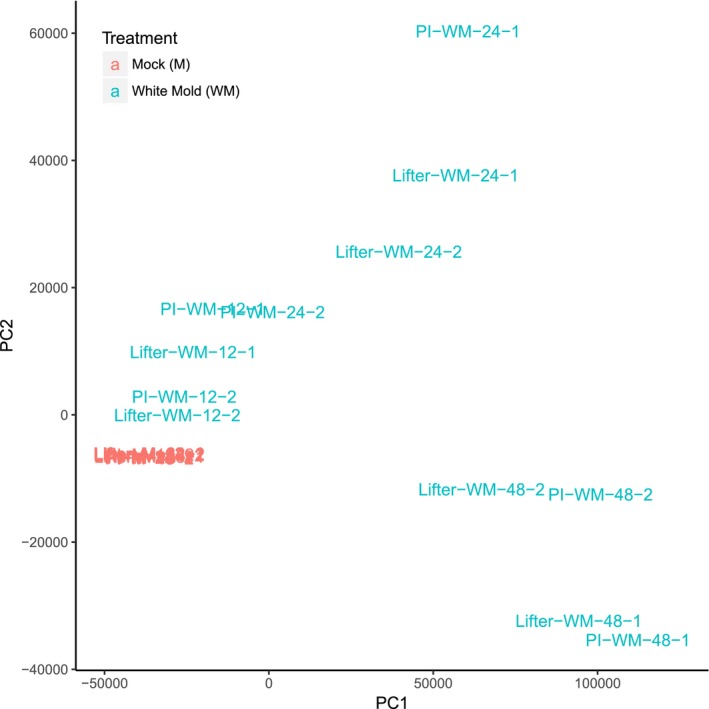
Principal component analysis (PCA). PCA defines the distribution of samples in an orthogonal system that maximizes variance explanation in the first and the second principal components (PC). PC1 explains about 75% of total variance from the RNA‐Seq gene expression among samples based on the normalized transcripts per million, and it is correlated with white mold (WM) inoculation as the plot clearly indicates separation of samples in the first axis. As mock inoculated samples are grouped tightly to each other, it is clear WM inoculation was the major influencing factor on gene expression. For *Sclerotinia sclerotiorum*‐inoculated samples, the time points appeared to be the second most influential factor

In order to understand the gene expression difference of the susceptible ‘Lifter’ and the partially resistant PI 240515 especially for those candidate resistance transcripts found in GWAS, the paired‐end reads of each sample were pseudo‐aligned to the de novo transcriptome containing 60,598 transcripts using Kallisto. A total of 17,220 genes with at least 5 estimated counts were found in 47% of samples, and these transcripts were kept for PCA. The expressions were normalized using TPM approach and quantified by Sleuth (Bray et al., 2017). *S. sclerotiorum* inoculation appeared to be the strongest influential factor to explain the variations of gene expression and the first principal component explained about 75% of variance (*x* axis). The treatments separated samples to two different spaces. Mock inoculated samples were clustered in one spot regardless of the pea lines and the time points, meaning relatively similar expression patterns (Figure 2). For *S. sclerotiorum*‐inoculated samples, the time points appeared to be the second most influential factor as samples from the same time point grouped close to each other.

### 3.6 Differential expression, heatmap clustering, and GO analysis

**Figure 3:**
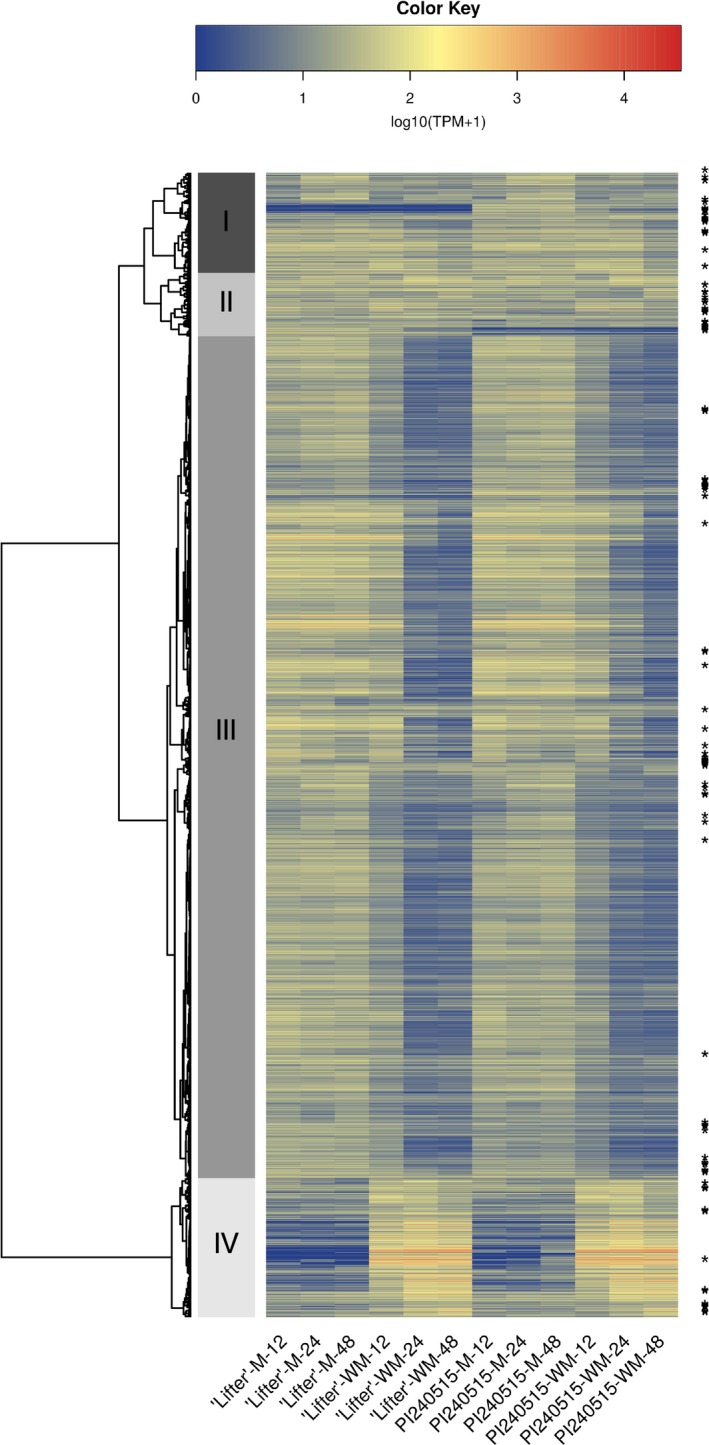
Heatmap and clustering analysis for differential expression (DE) transcripts over time. Transcripts with an asterisk have significant DE between ‘Lifter’ and PI 240515 only in *Sclerotinia sclerotiorum*‐inoculated samples but not mock samples. Clustering analysis breaks the 17,220 transcripts into four clusters. Cluster III contains transcripts that are generally down‐regulated in *S. sclerotiorum*‐inoculated samples, and cluster IV contains transcripts that are up‐regulated in *S. sclerotiorum*‐inoculated samples. Although cluster IV has higher expression after *S. sclerotiorum* inoculation, a few transcripts displayed significantly higher expression in PI 240515 than ‘Lifter,’ indicating most of the transcripts in cluster IV may be involved in common responses to pathogen infection but not necessarily candidate resistance genes

**Figure 4:**
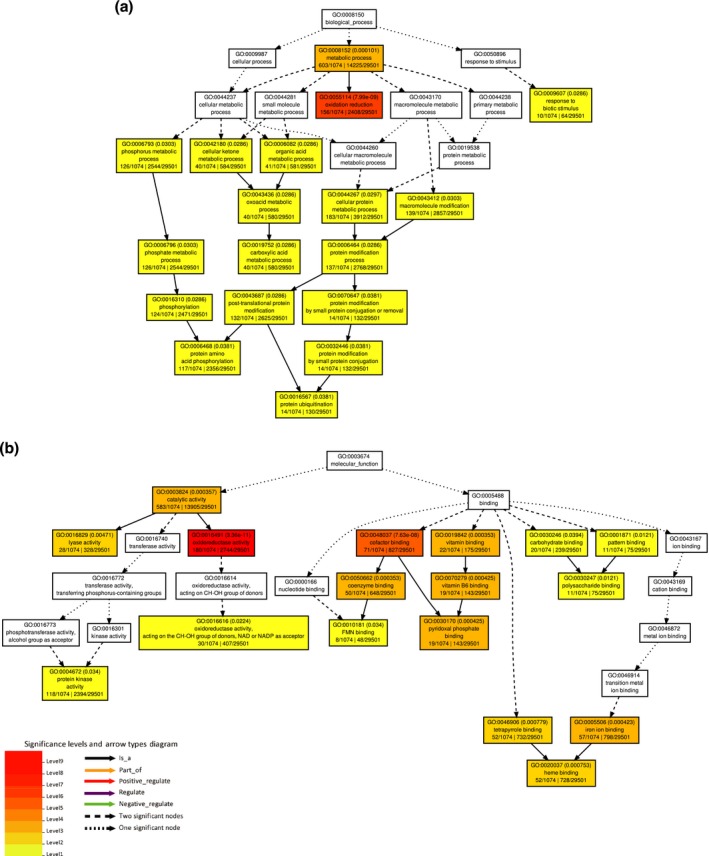
Gene ontology (GO) using singular enrichment analysis for transcripts in cluster IV. (a) Significant GO terms in the biological process. (b) Significant GO terms in the molecular functions. Color panel shows significant enrichment from level 1 in yellow color to level 9 in red color for both (a) and (b)

A total of 17,220 genes were analyzed for DE using a time series model. Transcripts were clustered into four groups based on their expression patterns in the heatmap (Figure 3, Supporting Information Table [Link], [Link]). While cluster III contains 12,668 transcripts that are generally down‐regulated, cluster IV contains 2,902 transcripts that are generally up‐regulated in the *S. sclerotiorum*‐inoculated samples regardless of the pea lines. On the other hand, cluster I and II, which contain 1,506 and 954 transcripts, respectively, do not have clear expression pattern differences in *S. sclerotiorum*‐inoculated samples compared to mock samples. While GO analysis using the SEA approach identified general biological process, cellular component, and molecular function for transcripts in clusters I, II, and III (Supporting Information Figures [Link], [Link], [Link], Supporting Information Table [Link], [Link]), transcripts in cluster IV were significantly enriched for oxidation reduction in the biological process (GO0055114, FDR: 7.99 × 10^−9^) and oxidoreductase activity in the molecular function (GO0016491, FDR: 3.36 × 10^−11^). These results indicated that many transcripts highly induced in cluster IV after *S. sclerotiorum* inoculation were related to redox maintenance (Figure 4a). Other than transcripts with potential redox balancing functions, significant enrichment for transcripts with cofactor‐, vitamin‐, heme‐, or iron‐binding functions were also found (Figure 4b). Because it has been suggested that oxalic acid stimulates iron release and soybeans were shown to express higher ferritin for capturing iron and maintaining iron homeostasis during infection (Calla, Blahut‐beatty, Koziol, Simmonds, & Clough, 2014), the enrichment results of these element‐binding transcripts in pea may indicate the homeostasis struggle during infection. Although most transcripts in cluster IV had higher expression after *S. sclerotiorum* inoculation, only a few transcripts displayed significantly higher expression in PI240515 than ‘Lifter,’ and together with the results from GO analysis, the possibilities that transcripts in cluster IV are genes involved in common responses to pathogen infection could not be excluded.

**Figure 5:**
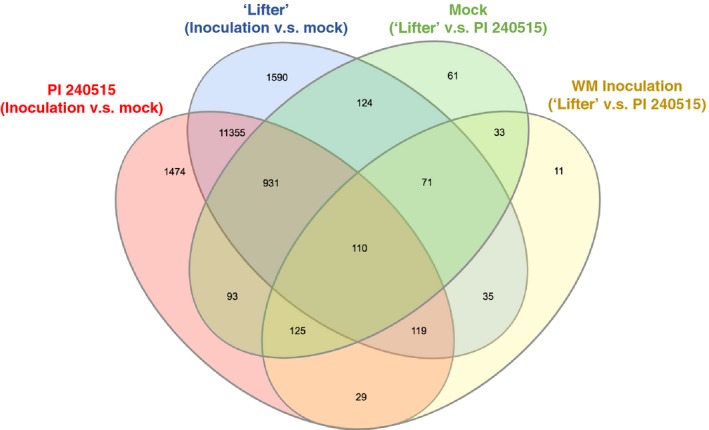
Venn diagram comparisons of time series differential expression analyses. In green, transcripts with significant DE between ‘Lifter’ and PI 240515 in mock samples. In yellow, transcripts with significant DE between ‘Lifter’ and PI 240515 in *Sclerotinia sclerotiorum*‐inoculated samples. In purple, transcripts of ‘Lifter’ with significant DE between mock samples and *S. sclerotiorum*‐inoculated samples. In pink, transcripts of PI 240515 with significant DE between mock samples and *S. sclerotiorum*‐inoculated samples. To narrow the candidate resistance genes pool from all DE, two assumptions were made: (i) a candidate gene should respond to *S. sclerotiorum* inoculation, and (ii) the expression of a candidate gene should have up‐regulated and significant DE in the *S. sclerotiorum*‐inoculated PI 240515 compared to the *S. sclerotiorum*‐inoculated ‘Lifter’ samples, but not the mock samples of PI 240515 compared to the mock samples of ‘Lifter’

**Figure 6:**
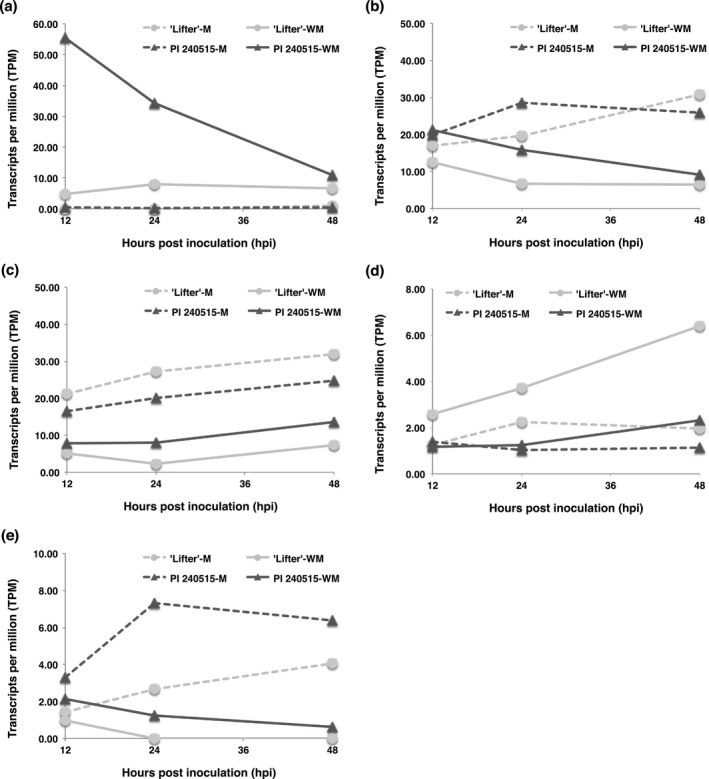
Time course expressions of LRR‐RLK transcripts identified from differential expression analyses. M represents mock samples, and WM represents *Sclerotinia sclerotiorum*‐inoculated samples. (a) TRINITY_DN22904_c0_g1_i2 (b) TRINITY_DN23231_c0_g2_i2 (c) TRINITY_DN18054_c0_g1_i1 (d) TRINITY_DN4777_c0_g1_i1 (e) TRINITY_DN21848_c0_g1_i1

To identify candidate genes involving *S. sclerotiorum* resistance using RNA‐Seq results (Supporting Information Tables [Link], [Link], [Link], [Link]), two assumptions were made: (i) a candidate gene should respond to *S. sclerotiorum* inoculation, and (ii) the expression of a candidate gene should have significant DE in the *S. sclerotiorum*‐inoculated samples of PI 240515 compared to the *S. sclerotiorum*‐inoculated samples of ‘Lifter,’ but not the mock samples of PI 240515 compared to the mock samples of ‘Lifter.’ A Venn diagram was illustrated to indicate the number of DE transcripts for four different comparisons (Figure 5, Supporting Information Table S10). Two of these sections fulfill our assumptions. The first contains 119 transcripts, which is the overlapping area among the blue (transcripts of ‘Lifter’ with DE in *S. sclerotiorum*‐inoculated samples compared to mock samples), red (transcripts of PI 240515 with DE in *S. sclerotiorum*‐inoculated samples compared to mock samples), and yellow blocks (transcripts of *S. sclerotiorum*‐inoculated samples with DE in PI 240515 compared to ‘Lifter’) but not the green block (transcripts of mock samples with DE in PI 240515 compared to ‘Lifter’) (Supporting Information Table S11). The second section which fulfills the assumptions contains 29 transcripts, which corresponds to the overlapping area of the red and yellow blocks (transcripts with DE in PI 240515 but not ‘Lifter,’ and these transcripts had DE in PI 240515 compared to ‘Lifter’ under *S. sclerotiorum* inoculation) (Supporting Information Table S12). While most of these transcripts (119 + 29 transcripts) had lower expression in PI 240515 compared to ‘Lifter’ after *S. sclerotiorum* inoculation, a few transcripts had higher expression in PI 240515 than ‘Lifter’ after *S. sclerotiorum* inoculation, including three transcripts that encode LRR receptor‐like kinase (LRR‐RLK). The first LRR‐RLK is TRINITY_DN22904_c0_g1_i2, which had nearly zero expression in mock samples, but the expressions were induced higher in PI 240515 than ‘Lifter’ after *S. sclerotiorum* inoculation (Figure 6a). The expressions of TRINITY_DN23231_c0_g2_i2 was higher in PI 240515 than ‘Lifter’ after *S. sclerotiorum* inoculation, but their expressions were down‐regulated after *S. sclerotiorum* inoculation compared to mock samples (Figure 6b). On the other hand, the expression of TRINITY_DN4777_c0_g1_i1 was generally higher in ‘Lifter’ than PI 240515, and *S. sclerotiorum* inoculation caused up‐regulation more in ‘Lifter’ than PI 240525 (Figure 6d) and the expression of TRINITY_DN18054_c0_g1_i1 and TRINITY_DN21848_c0_g1_i1 were higher in mock samples than in *S. sclerotiorum‐inoculated* samples (Figure 6c,e). It is worth noticing that the power for finding candidate resistance genes solely using expression difference is limited, and although the assumptions narrowed down the candidate pool, they may be subjective and other important genes might be neglected. Accordingly, we combined the power of GWAS and RNA‐Seq to search candidate resistance genes with both genetic mapping and expression evidence.

### 3.7 Integration of GWAS and RNA‐Seq results

**Figure 7:**
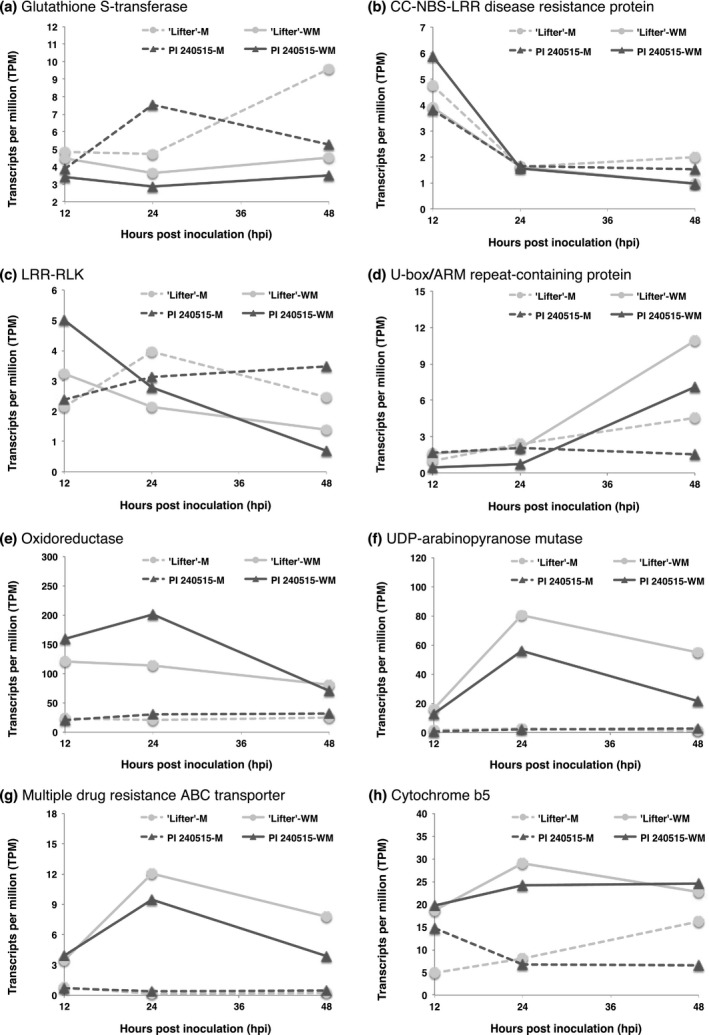
Time course expressions of candidate lesion resistance transcripts identified from both differential expression analyses and genome‐wide association studies. M represents mock samples, and WM represents *Sclerotinia sclerotiorum*‐inoculated samples. (a) TRINITY_DN7903_c0_g1_i2 (b) TRINITY_DN11274_c0_g2_i1 (c) TRINITY_DN21987_c1_g2_i1 (d) TRINITY_DN21727_c0_g1_i1 (e) TRINITY_DN15345_c0_g1_i2 (f) TRINITY_DN12885_c0_g1_i1 (g) TRINITY_DN23674_c1_g2_i1 (h) TRINITY_DN29578_c0_g1_i1

**Figure 8:**
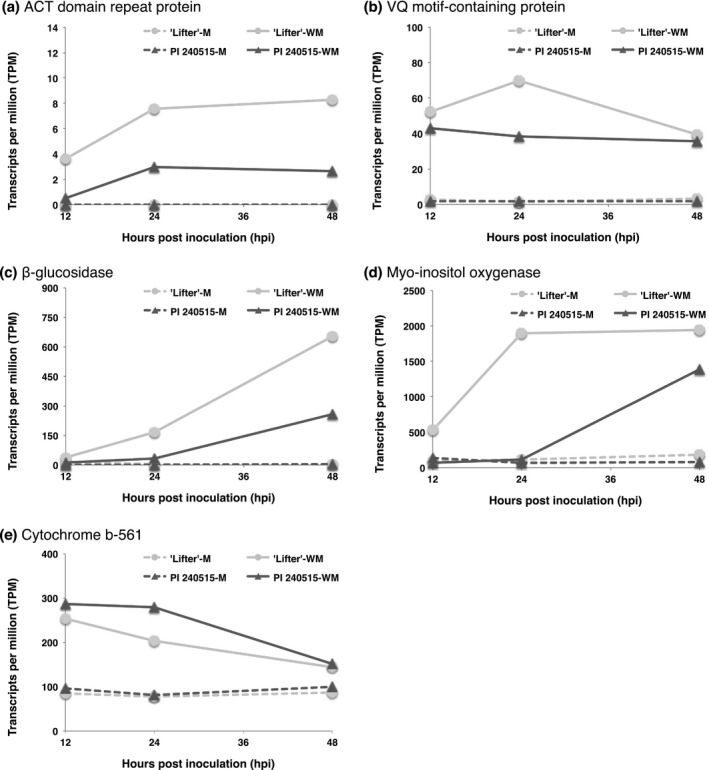
Time course expressions of candidate nodal resistance transcripts identified from both differential expression analyses and genome‐wide association studies. M represents mock samples, and WM represents *Sclerotinia sclerotiorum*‐inoculated samples. (a) TRINITY_DN5298_c0_g1_i1 (b) TRINITY_DN25769_c0_g1_i1 (c) TRINITY_DN23515_c1_g1_i4 (d) TRINITY_DN21524_c0_g1_i1 (e) TRINITY_DN16214_c1_g2_i1

Integration of results from DE analyses and GWAS identified additional candidate resistance genes; however, most transcripts were down‐regulated after *S. sclerotiorum* inoculation (Figure 3), and only a few transcripts had significantly higher expression in PI 240515 than ‘Lifter’ (Figures 7 and 8). The transcript (TRINITY_DN7903_c0_g1_i2) found for both lesion and nodal resistance, which encodes a putative GST, had DE after *S. sclerotiorum* inoculation (Figure 7a; Supporting Information Figure S4). There were two LRR‐containing DE transcripts (TRINITY_DN11274_c0_g2_i1 and TRINITY_DN21987_c1_g2_i1) that significantly associated with lesion resistance (Table 2; Figure 7b,c). Additionally, a DE transcript annotated as an U‐box/ARM repeat superfamily protein (TRINITY_DN21727_c0_g1_i1), an oxidoreductase (TRINITY_DN15345_c0_g1_i2), an UDP‐arabinopyranose mutase (TRINITY_DN12885_c0_g1_i1), a multiple drug resistance ABC transporter (TRINITY_DN23674_c1_g2_i1), and a cytochrome b5 (TRINITY_DN29578_c0_g1_i1) were all significantly associated with lesion resistance (Table 2; Figure 7d–g). On the other hand, there were five DE transcripts annotated as an ACT domain repeat protein (TRINITY_DN5298_c0_g1_i1), a VQ motif‐containing protein (TRINITY_DN25769_c0_g1_i1), a β‐glucosidase (TRINITY_DN23515_c1_g1_i4), a myo‐inositol oxygenase (TRINITY_DN21524_c0_g1_i1), and a cytochrome b‐561 (TRINITY_DN16214_c1_g2_i1) that were significantly associated with nodal resistance (Figure 8a–e; Supporting Information Figure S4). Among these transcripts, a putative coiled‐coil nucleotide‐binding site leucine rich repeat (CC‐NBS‐LRR) protein appeared interesting as a lesion resistance candidate because its expression was up‐regulated in PI 240515 but down‐regulated in ‘Lifter’ after 12 hpi (Figure 7b). As for nodal resistance, only the putative cytochrome b‐561 had higher expression in PI 240515 than ‘Lifter,’ and other transcripts mostly had higher DE in ‘Lifter’ than PI 240515 (Figure 8e).

## 4 DISCUSSION

### 4.1 Redox homoeostasis is important for both lesion and nodal resistance

In this study, we aimed to understand the genetic makeup of lesion and nodal resistance in pea for resistance to *S. sclerotiorum*. Although the weak phenotypic correlation between stem lesion and nodal resistance indicated the likelihood of distinct genetic makeups for lesion and nodal resistances, GWAS identified one SNP (C/T), TP13557, for both phenotypic ratings. The SNP was mapped to a transcript that encodes for a putative glutathione S‐transferase (GST) of pea. Interestingly, GST of corn (*Zea mays*) has been identified as a pleiotropic resistance gene for three fungal diseases, southern leaf blight (caused by *Cochliobolus heterostrophus*), gray leaf spot (caused by *Cercospora* species), and northern leaf blight (caused by *Setosphaeria turcica*) in a multivariate mapping study (Wisser et al., 2011). While the significant SNP located in the 3′‐UTR of corn GST, three nonsynonymous substitutions were found in the coding sequence, and one of them (histidine to aspartic acid) may contribute about 6% of resistance (Wisser et al., 2011). Other studies also pointed out the importance of GST in potato, rice, and tobacco to *Phytophthora infestans*,* Magnaporthe oryzae*, and two *Colletotrichum* species, respectively (Dean, Goodwin, & Hsiang, 2005; Leonards‐Schippers et al., 1994; Wisser, Sun, Hukbert, Kresovich, & Nelson, 2005). Moreover, most studies focusing on *B. napus* resistance to *S. sclerotiorum* also identified GST regardless of the methodologies, GWAS or RNA‐Seq (Girard et al., 2017; Wei et al., 2016; Wu, Zhao, Liu, et al., 2016). A recent GWAS for soybean resistance to *S. sclerotiorum* also identified soybean GST (Wei et al., 2017) which was also noted to have high expression in a transcriptomic study (Calla et al., 2014). Consistently, our results also pointed out GST of pea may play a fundamental role in lesion and nodal resistance to *S. sclerotiorum*. GST has diverse molecular functions in a cell to balance redox homoeostasis, and glutathione is important for maintaining a reducing status for cell survival (Tew, 2007). Accordingly, GST may be involved in prohibiting the switch from biotrophic to necrotrophic stage of *S. sclerotiorum*. Another GST function is to detoxify phytotoxins and oxidative substances such as ROS (Wisser et al., 2011), and these functions may slow *S. sclerotiorum* infection and plant cell death. Other than GST, redox‐related genes were up‐regulated after *S. sclerotiorum* inoculation and were significantly enriched in cluster IV, and redox‐related enzymes such as an oxidoreductase and a cytochrome b‐561 were found in GWAS for lesion or nodal resistance, respectively. Although the molecular function of cytochrome b‐561 in plant resistance is not clear, it has been also discovered for *S. sclerotiorum* resistance in *B. napus* using GWAS and RNA‐Seq (Wei et al., 2016). Surprisingly, our combined RNA‐Seq and GWAS strategy to search for *S. sclerotiorum* resistance in pea ended up with results similar to the study of *B. napus*, where β‐glucosidase, TPR‐containing protein, ARM repeat superfamily protein, cytochrome b‐561, LRR‐containing proteins, and the GST were also found for *B. napus* (Supporting Information Tables S3 and S4; Wei et al., 2016). Together, our results support the importance of redox homoeostasis for *S. sclerotiorum* resistance, and we identified many potential redox‐related transcripts as well as others with roles in basal resistance to white mold.

### 4.2 Lesion resistance

Several LRR‐containing genes were found in transcriptomic studies of *B. napus*‐*S. sclerotiorum* (Wei et al., 2016; Wu, Zhao, Yang, et al., 2016). The results of our study also discovered several up‐regulated LRR‐containing transcripts for lesion resistance but not nodal resistance. Two transcripts with evidence from both GWAS and RNA‐Seq are a putative CC‐NBS‐LRR protein and an LRR‐RLK protein (Figure 7b,c). Both transcripts had higher expression in PI 240515 at 12 hpi but the expressions dropped over time to the expression level of ‘Lifter.’ Although it is well known that LRR‐containing proteins contribute to R‐gene based resistance in plants to biotrophic pathogens (Kushalappa, Yogendra, & Karre, 2016), it is unclear how much these LRR‐containing transcripts are involved in lesion resistance to *S. sclerotiorum*. Moreover, these LRR‐containing transcripts were not discovered in GWAS for nodal resistance, which indicated the possibility that the lesion resistance relies on LRR‐containing proteins more than nodal resistance. Other than the LRR type of tandem repeats, several U‐Box/ARM repeat‐containing proteins were found for lesion resistance by GWAS (Table 2, Supporting Information Table S3). It has been shown that an U‐Box/ARM‐containing ligase in rice negatively controls resistance (Li et al., 2012; Sharma & Pandey, 2015; Zeng et al., 2004), indicating higher expression in ‘Lifter’ might favor *S. sclerotiorum* infection (Figure 7d). While plant cell wall synthesis enzymes such as cellulose synthase were identified, only a putative UDP‐arabinopyranose mutase was up‐regulated after *S. sclerotiorum* inoculation (Figure 7f). Similarly, two pleiotropic drug resistance ABC transporters were found (Table 2) but only one displayed up‐regulation after *S. sclerotiorum* inoculation (Figure 7g). It has been shown that a pleiotropic drug resistance ABC transporter is involved in resistance to *Botrytis cinerea*, a closely‐related fungal species to *S. sclerotiorum* (Stukkens et al., 2005). Accordingly, our results suggested diverse mechanisms were involved in lesion resistance to limit *S. sclerotiorum* expansion.

### 4.3 Nodal resistance

Five transcripts found by both GWAS and RNA‐Seq had higher DE after *S. sclerotiorum* inoculation (Figure 7). While the ACT domain repeat‐containing proteins have diverse functions in plant physiologies (Feller, Yuan, & Grotewold, 2017), the β‐glucosidase might be involved in cell wall reinforcement or releasing damage associated molecular patterns (DAMP) (Duran‐Flires & Heil, 2016). Additionally, one of the transcripts identified for nodal resistance is a putative VQ motif‐containing protein, which had higher expression in response to *S. sclerotiorum* infection (Figure 8b). It has been shown that two VQ motif‐containing proteins, VQ12 and VQ29, in *Arabidopsis* negatively regulate resistance to *B. cinerea*. Down‐regulation of VQ12 and VQ29 by miRNA silencing promoted *Arabidopsis* resistance to *B. cinerea* while overexpression increased susceptibility (Wang, Hu, Pan, & Yu, 2015). In addition, overexpression of *Arabidopsis* VQ5 and VQ20 demonstrated enhanced susceptibility to *B. cinerea* and *Pseudomonas syringae* (Cheng et al., 2012). These discoveries might help to explain the potential functions of this VQ motif‐containing protein in pea‐*S. sclerotiorum* interaction.

Another transcript with earlier and higher expression in ‘Lifter’ was a putative myo‐inositol oxygenase (Figure 7d). Myo‐inositol is a product catalyzed from glucose‐6‐phosphate by the myo‐inositol‐1‐phosphate synthase, and it can be further metabolized into UDP‐glucuronic acid by myo‐inositol oxygenase (Kanter et al., 2005). One of the functions of UDP‐glucuronic acid is being the precursor of plant cell wall polysaccharides, and under the circumstance, earlier and higher expression of this transcript in ‘Lifter’ may indicate the need of plant cell wall reinforcement under high *S. sclerotiorum* pressure. Gene expression difference for myo‐inositol metabolism was also reported in resistant and susceptible soybeans to *S. sclerotiorum* (Calla et al., 2009). Additionally, myo‐inositol is also the precursor of galactinol, which has been suggested to induce resistance against syncytia development for the cyst nematode *Heterodera schachtii* (Siddeigue et al., 2014). When myo‐inositol oxygenase processes myo‐inositol into UDP‐glucuronic acid, the metabolism bypasses and reduces the production of galactinol for inducing disease resistance signaling (Cho et al., 2010; Kim et al., 2008). Under this scenario, disease progress in ‘Lifter’ could be faster than in PI 240515. Because *S. sclerotiorum* can still infect PI 240515 at a slower progression than ‘Lifter,’ it is possible that lower expression of transcripts favoring *S. sclerotiorum* infection underlies nodal resistance in PI 240515. More studies are needed to reveal the molecular mechanisms of nodal resistance.

### 4.4 Summary

In this study, we localized significant SNPs identified from GWAS using a de novo transcriptome. We also incorporated expression analyses to understand the responses of candidate resistance genes to *S. sclerotiorum* infection. While we revealed SNPs exclusively for either lesion or nodal resistance, it is worth noticing that many SNPs appear to locate in the intergenic regions of the pea genome and many de novo transcripts might be too short to be annotated perfectly. The availability of a pea reference genome will not only improve the precision of SNP analysis but also provide a comprehensive understanding on coding sequences of pea. Nonetheless, our strategy integrating the advantages of GWAS and RNA‐Seq indicated that aside from a single SNP located within a transcript encoding GST, there are likely different genetic mechanisms underlying lesion and nodal resistance.

## ACCESSION NUMBER

The Illumina sequences were deposited at SRA database under BioProject accession number PRJNA261444.

## CONFLICT OF INTEREST

The authors claimed no conflict of interest.

## AUTHOR CONTRIBUTIONS

H.‐X.C., H.S., X.Z., and L.D.P. conducted plant inoculation and phenotyping. H.‐X.C, H.S., and J.W. performed data analyses. K.E.M., L.D.P., and M.I.C. maintained plant and fungal materials. X.Z. generated the RNA‐Seq data. H.‐X.C. and M.I.C. led the manuscript writing, and all the co‐authors refined and approved the manuscript.
