# Supplementary figures and images for: Exploring the genetics of lesion and nodal resistance in pea (Pisum sativum L.) to Sclerotinia sclerotiorum using genome‐wide association studies and RNA‐Seq
Source: Plant Direct. 2018 Jun 26;2(6):e00064. doi: 10.1002/pld3.64 (PMC6508546; doi:10.1002/pld3.64)

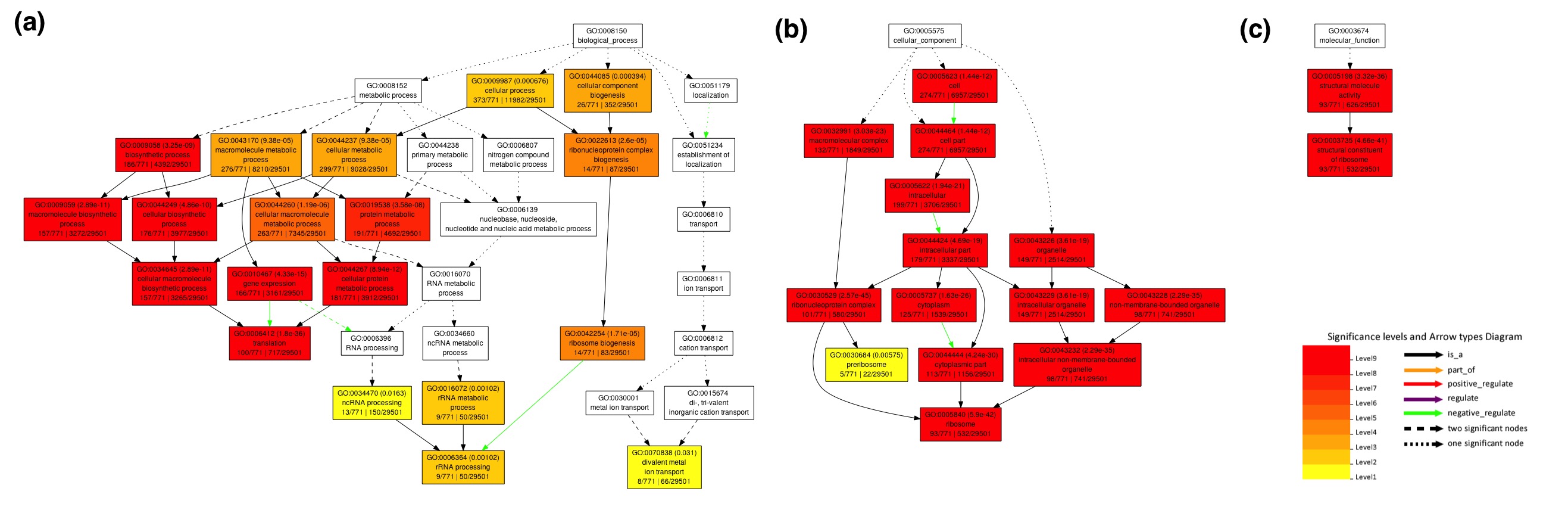

Supplement: Supplementary file 1 [file PLD3-2-e00064-s001.jpg]

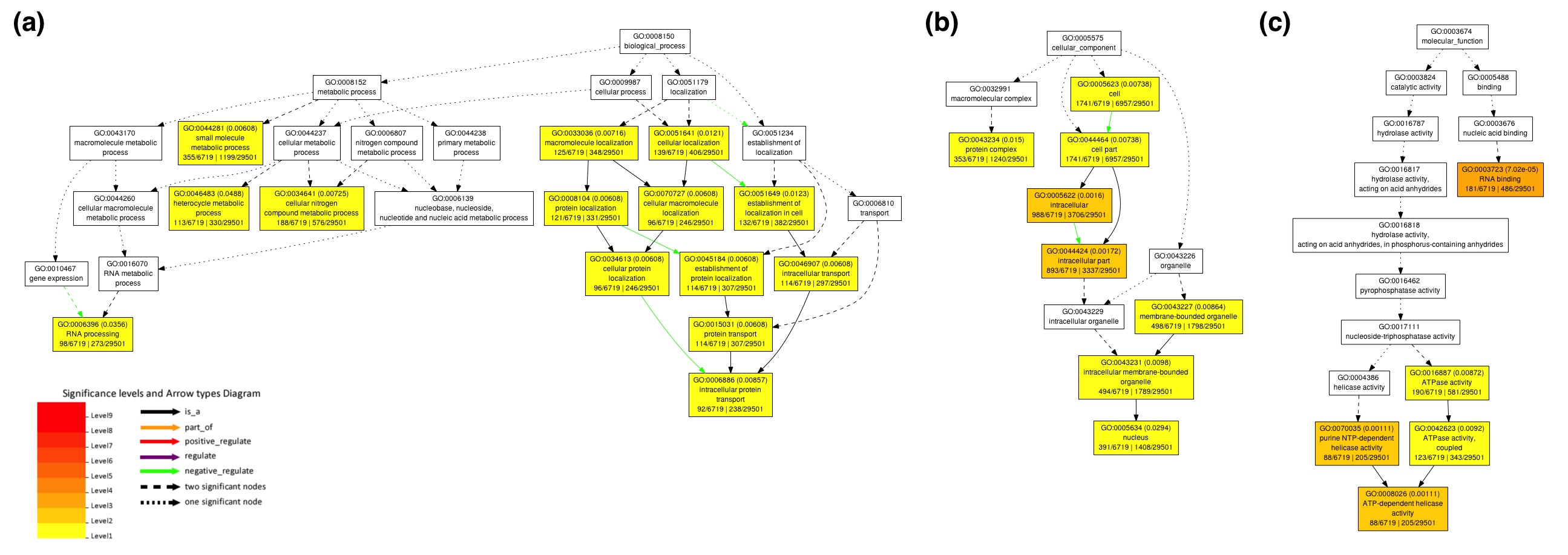

Supplement: Supplementary file 2 [file PLD3-2-e00064-s002.jpg]

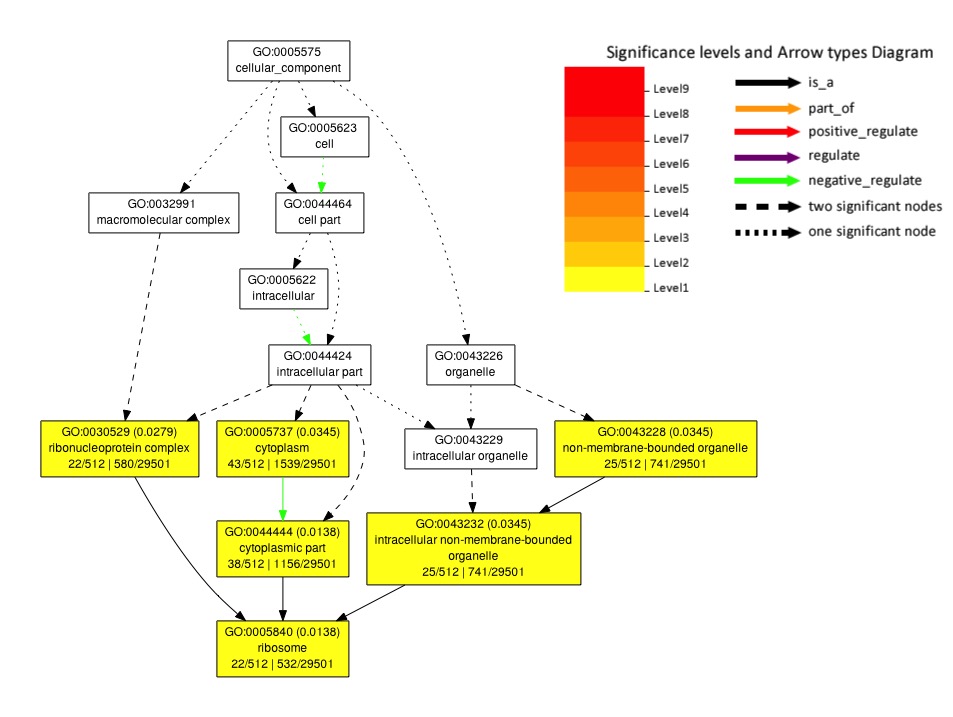

Supplement: Supplementary file 3 [file PLD3-2-e00064-s003.jpg]

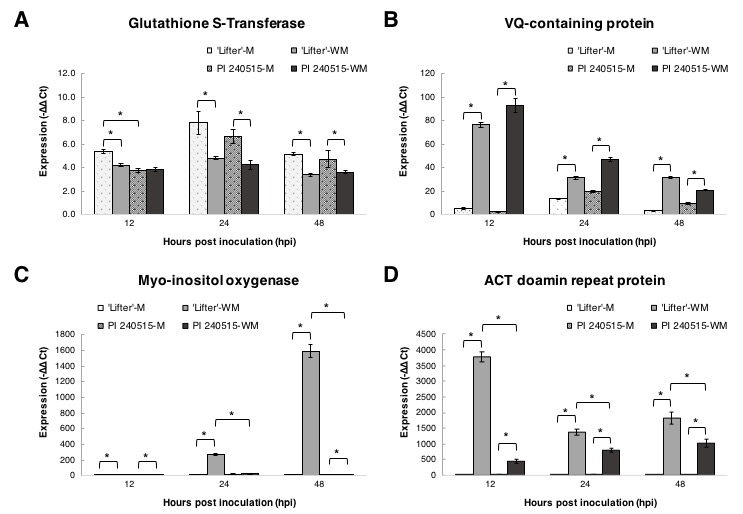

Supplement: Supplementary file 4 [file PLD3-2-e00064-s004.jpg]
