# Supplementary material for: Exploring the genetics of lesion and nodal resistance in pea (Pisum sativum L.) to Sclerotinia sclerotiorum using genome‐wide association studies and RNA‐Seq
Source: Plant Direct. 2018 Jun 26;2(6):e00064. doi: 10.1002/pld3.64 (PMC6508546; doi:10.1002/pld3.64)
Supplement: Supplementary file 18 [file PLD3-2-e00064-s018.pdf]

Re: submission 2017-00111

Dear Gaurav Moghe and Ana Fortes,

Thank you for your comments and those of reviewer 3. We have addressed your requests on expression verification in the manuscript and provided a point by point response below. Included below are the first and second round review comments. Please let us know if you have any concerns.

Regards,

Hao-Xun Chang, Martin Chilvers and co-authors.

Responses to Editors' Comment

Based on Comment #1 from Reviewer #3, we request that additional validation of the expression levels be performed using either qPCR or semi-quantitative RT-PCR. We understand that doing so for all the genes in Figs. 7 and 8 is not feasible and also not required. Validation may be performed for 4 genes as described in section 3.7 (Integration of GWAS and RNA-seq results), using an appropriate internal standard and preferably including 3 biological replicates instead of 2 (if the two biological replicates used in RNAseq come from a pool of more biological replicates collected separately). We believe that Reviewer #3's comments will significantly bolster confidence in the final result. The authors do not need to respond to Comment #2 from Reviewer #3.

Response to second round of review: Thank you for the comments. We followed your suggestions and performed a RT-qPCR on four genes mentioned in section 3.7. We chose a recommended reference gene for pea under biotic stress (Die et al. 2010), and the RT-qPCR was performed with two technical replicates for each of the three biological replicates. RT-qPCR confirmed these genes were differentially expressed in response to *Sclerotinia sclerotiorum* infection, and the methods and the results were added to the manuscript.

Responses to Reviewers' Comment

Reviewer #3 :

Most of the concerns have been addressed. But, I highly recommend publication of the manuscript after considering the following two major revision.

1. Please validate the transcripts of figure 7and 8 with RTPCR.

Response to first round of review: We do not see the necessity of RT-qPCR for verifying RNA-Seq data. RNA-Seq is now regarded more reliable than RT-qPCR. RT-qPCR relies on one or a couple of housekeeping genes as a reference, but the searching of housekeeping genes and the validity of housekeeping genes (e.g. tubulin or actin) for their stability over tissues, genotypes, time points, and treatments required intensive confirmation. Instead, RNA-Seq normalizes counts of each transcript against all transcripts in the transcriptome. In addition, RT-qPCR relies on the assumption of doubling amplification for each cycle but amplification frequency for each primer set may be different. Amplification of a 100-200 bp fragment may or may not represent the transcription level of a full length of mRNA, especially if the gene is larger such as 2 Kb. RNA-Seq has better coverage and counts raw reads that match to a transcript at any possible location instead of a subjective region chosen for primer design.

Reviewer comment - RNA-seq is a highly automated procedure for global gene expression profiling which may not exactly reflect the expression status of all genes. The data obtained in RNAseq sensitivity is also dependent on read depth (whether it is 50 M reads or 100 M reads) or the length of the targeted genome.
Moreover, if you have look at your RNA-Seq read mapping. The read coverage depth could vary between different positions on your candidate gene. Your FPKM values are computed

by considering all reads mapped to your gene "A". It could be possible that the specific position shows a 0.5-fold difference while the overall difference is 2-fold.
I would suggest authors read these articles which will help them understand, why validation of RNA seq data is required and how you can perform better RTPCR.
https://onlinelibrary.wiley.com/doi/abs/10.1111/tpj.13014
http://www.plantcell.org/content/20/7/1736
The overall study by authors have led to identifying few genes and then there is no functional study done, it becomes important to validate them using qPCR. qPCR is highly standardized procedure but RNAseq procedure and analysis is still evolving. I would highly recommend qPCR to be done to validate the data using internal control genes. I agree with the author that internal genes chosen can have a problem too, for this I would suggest the author look at their NGS data and find the internal control which does not change much during the study and use them for qPCR.
If primers are carefully designed, amplifying the only specific gene and the amplification efficiency is high, RT-PCR should be able to provide the most accurate abundance of a particular target in a gene expression study.

Response to second round of review: We thank the reviewer for their suggestions, and RT-qPCR references. We have included RT-qPCR, see Supplementary Figure 4. Although there were differences in the magnitude of responses between the RNAseq and the RT-qPCR data, the trends of expression between the data sets concurred. GST was down regulated upon inoculation with *S. sclerotiorum*. While the ACT domain repeat protein, VQ motif-containing protein and myo-inositol oxygenase were up regulated upon inoculation with *S. sclerotiorum*.

2. Fig. 2. Sample PIWM 24-1 and 2 is behaving very differently one of them is clustering with 12-hour time point while other is way different than all other samples. Which might contribute to the pseudo conclusion about transcriptomics data, I would suggest one more replicate to be performed.

Response to first round of review: Although the variation between PI-WM-24-1 and PI-WM-24-2 are larger than other samples, the difference did not invert the observation that WM

inoculation is the largest factor determining expression variation. Accordingly, statistical tests on identifying transcripts responded to WM inoculation should be valid. Additional RNA-Seq is beyond our current scope.

Reviewer comment - If additional RNA seq is not doable I would suggest removing 24-hour data point study. As time series study will become difficult to conclude with such large variation at 24 hours between susceptible and resistant response.

Response to second round of review: The PCA shows the first two large variables in a two dimensional layout, which provides an intuitive understanding between the variance sources and experimental setups. We understand the reviewer's concern on the distribution difference between PI-WM-24-1 and PI-WM-24-2, but there is no statistical evidence to show these two samples were outliers. Their distribution did not invert the trend that 1st PC separates *S. sclerotiorum* inoculation and 2nd PC separates time points. The mixture distribution of *S. sclerotiorum*-inoculated susceptible and resistant cultivars could be observed in 12 dpi and 48 dpi as well, and it is possible their distributions (including PI-WM-24-1 and PI-WM-24-2) will appear closer in other PC dimensions. The RNA-Seq part of our study aimed to identify differential expressed genes in response to *S. sclerotiorum* infection, in order to provide gene expression insights in the integration with genetic mapping for lesion and nodal resistance.
